# Cumulative Incidence of Venous Thromboembolic Events In-Hospital, and at 1, 3, 6, and 12 Months After Metabolic and Bariatric Surgery: Systematic Review of 87 Studies and Meta-analysis of 2,731,797 Patients

**DOI:** 10.1007/s11695-024-07184-7

**Published:** 2024-04-11

**Authors:** Walid El Ansari, Ayman El-Menyar, Kareem El-Ansari, Abdulla Al-Ansari, Merilyn Lock

**Affiliations:** 1 Department of Surgery, Hamad Medical Corporation, 3050 Doha, Qatar; 2 College of Medicine, Qatar University, Doha, Qatar; 3 Department of Clinical Population Health, Weill Cornell Medicine-Qatar, Doha, Qatar; 4 Clinical Research, Trauma and Vascular Surgery, Hamad Medical Corporation, Doha, Qatar; 5 Department of Clinical Medicine, Weill Cornell Medicine-Qatar, Doha, Qatar; 6 Faculty of Medicine, St. George’s University, Saint George’s, Grenada; 7 Department of Exercise Science, Health and Epidemiology, College of Health and Life Sciences, Hamad Bin Khalifa University, Doha, Qatar

**Keywords:** Systematic review, Meta-analysis, Morbid obesity, Bariatric surgery, Venous thromboembolism, Incidence, Laparoscopic procedure, Open surgery

## Abstract

**Abstract:**

Systematic review/meta-analysis of cumulative incidences of venous thromboembolic events (VTE) after metabolic and bariatric surgery (MBS). Electronic databases were searched for original studies. Proportional meta-analysis assessed cumulative VTE incidences. (PROSPERO ID:CRD42020184529). A total of 3066 records, and 87 studies were included (N patients = 4,991,683). Pooled in-hospital VTE of mainly laparoscopic studies = 0.15% (95% CI = 0.13–0.18%); pooled cumulative incidence increased to 0.50% (95% CI = 0.33–0.70%); 0.51% (95% CI = 0.38–0.65%); 0.72% (95% CI = 0.13–1.52%); 0.78% (95% CI = 0–3.49%) at 30 days and 3, 6, and 12 months, respectively. Studies using predominantly open approach exhibited higher incidence than laparoscopic studies. Within the first month, 60% of VTE occurred after discharge. North American and earlier studies had higher incidence than non-North American and more recent studies. This study is the first to generate detailed estimates of the incidence and patterns of VTE after MBS over time. The incidence of VTE after MBS is low. Improved estimates and time variations of VTE require longer-term designs, non-aggregated reporting of characteristics, and must consider many factors and the use of data registries. Extended surveillance of VTE after MBS is required.

**Graphical Abstract:**

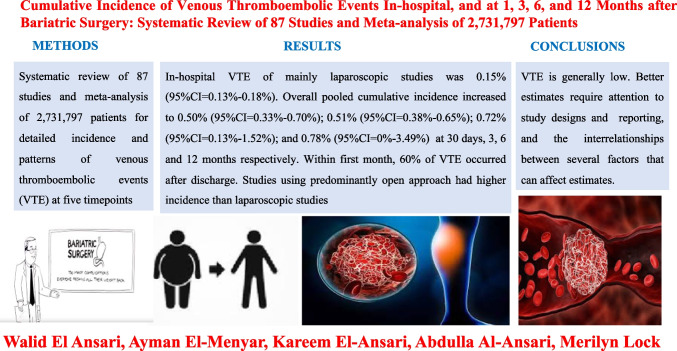

## Introduction

Metabolic and bariatric surgery (MBS) is an effective approach for achieving weight loss and resolution of obesity-associated medical problems among patients with obesity [1, 2]. As with any surgical procedure, there is the risk of post-operative venous thromboembolic events (VTE) after MBS, which is exacerbated by the underlying obesity [3]. With the increasing global frequency of MBS [4], VTE presents a particularly serious complication with significant effects on readmission rates and mortality [3, 5, 6].

The literature has shown wide variations in the incidence of VTE after MBS [7, 8]. For instance, previous studies have reported 30-day incidences ranging from 0 to 5.66% [9, 10]. Although the number of meta-analyses on many MBS topics continues to increase [11–13], there are no systematic reviews/meta-analyses of the incidence of VTE after MBS. This is despite the available literature that could be meta-analyzed to generate high-quality estimates [5, 7, 10, 14–17]. To date, global estimates of VTE at different timepoints after MBS remain uncertain, despite the calls for more accurate estimates [7]. The current study is the first to bridge this knowledge gap.

### Aim of the Study

The present study aimed to review and synthesize the evidence on the incidence of VTE after MBS. The objectives were to (1) compute global cumulative incidence of VTE at five timepoints after surgery (in-hospital, at 30 days and 3, 6, and 12 months) for studies that utilized mainly laparoscopic approach and those that used predominantly open surgical approach and (2) for the first 30 days investigate the proportions of VTE that occurred in-hospital vs post-discharge. In addition, we subgrouped the studies at each timepoint by two variables, namely, *geographic origin* and *study age* (final year of data acquisition) to explore potential sources of heterogeneity, and appraise whether such factors influenced the incidence of VTE. Evaluation of procedure- or patient-related risk factors or prophylaxis and their associations with VTE were not within the scope of this review.

## Materials and Methods

The Preferred Reporting Items for Systematic Reviews and Meta-Analyses (PRISMA) Statement was used to conduct and report this systematic review with meta-analysis [18]. The study protocol was registered a priori (PROSPERO ID: CRD42020184529).

### Search Strategy

A systematic search for relevant studies was conducted on 27 April 2022 using PubMed, MEDLINE, and Scopus. The full search strategy, including search terms and medical subject headings (MeSH), is detailed in Supplementary File 1. The reference lists of related reviews were checked for eligible studies that were not captured in the search.

### Study Selection

The inclusion criteria were original English language studies of any design, sample size, MBS procedure, or surgical approach that provided cumulative incidence of VTE or sufficient detail to calculate it for the total patient sample. Exclusion criteria included commentaries, letters, practice guidelines, reviews, and conference proceedings; studies that did not include VTE; studies that applied specific limitations to the patient populations they examined, e.g., specific age, weight or BMI cutoffs, obesity associated medical problems, or ethnicity; or questionnaire-based studies reporting subjective recall of VTE from clinicians. Studies were included if they accounted for all VTE including both deep vein thrombosis and pulmonary embolism. Studies that accounted only for specific sub-types of VTE were excluded.

### Screening and Data Extraction

Duplicate titles were removed and then all references were independently screened by two authors (WEA, ML) using Covidence (Veritas Health Innovation, Australia). The first stage was title and abstract screening, and studies were excluded if both authors rejected them. In the second stage, full-text articles were screened for eligibility, and studies approved by both authors were included. Conflicts were resolved through discussion between the two authors. Data extraction was undertaken and tabulated by WEA and KE-A and verified by ML and included study identifiers, country, sample size and characteristics, data sources, duration of data acquisition, surgical procedure, approach (laparoscopic, open, robotic), and the reported incidence and timing of VTE. Missing data were calculated where possible or extracted from figures using WebplotDigitizer 4.5 (Ankit Rohatgi, USA).

### Outcomes

The outcomes were cumulative incidence of VTE at five timepoints (in-hospital, 30 days, and 3, 6, 12 months) and for the first 30 days, the proportions of VTE that occurred in-hospital vs post-discharge.

### Risk of Bias Assessment

Potential risk of bias was assessed using the tool by Loney et al. [19], which was developed specifically for studies of prevalence/incidence. The tool was selected for its comprehensiveness and applicability to the study objectives. It comprises eight equally weighted items yielding a maximum score of eight, with higher scores indicating lower risk of bias. Included studies were scored by ML, and then 10% was randomly selected and scored by WEA. Mean percentage agreement across the eight individual items was reported.

### Data Synthesis and Statistical Analysis

All studies were cross-checked for duplicated use of data by verifying their data sources (hospital or national/regional registries), sampling timeframe, and included procedures. Where duplicate use of patient data was suspected, only the studies that minimized any overlap were included in the meta-analyses. As many of the included studies were undertaken using large administrative datasets such as NSQIP, MBSAQIP, or NIS, multiple studies included in the same year/s of data from the same registry were meticulously securitized for their procedures, patient samples, and recruitment years, in order to check, confirm, and exclude any potential duplicate use of the data of the same patients. If there was any remaining doubt, the research team undertook the extra step of contacting the authors of such papers for more verification.

Random effects proportional meta-analyses of VTE at the five timepoints were conducted using MetaXL (EpiGear, international Pty Ltd., Queensland, Australia) for Microsoft Excel. Data were transformed using the double arcsine method. This allows inclusion of zero-case studies, stabilizes variance, and has demonstrated advantages [20]. Additionally, categorical meta-analysis assessed pre- versus post-discharge 30-day incidence and was expressed as a proportion of the total number of cases.

Results were presented by surgical approach as pooling both (laparoscopic and open) approaches was deemed inappropriate because most procedures are currently undertaken laparoscopically. Most studies reported a mix of laparoscopic and open approaches; hence, cut-offs were required. As approximately half of the studies that used a majority open approach reported it for 50–80% of their procedures, and almost all studies that used a majority laparoscopic approach reported it for > 80% of their procedures, we subgrouped studies into “ > 80% laparoscopic approach” vs “ > 50% open approach.” Furthermore, subgroup analyses were conducted on cumulative incidences at each timepoint to identify any influence of the subgroups on the pooled estimates, and to assess sources of heterogeneity. In terms of study age, we categorized studies into those with data collected up to the end of 2010 vs after 2010, as an earlier cut-off was not feasible due to a lack of relevant studies. Geographically, it was only feasible to subgroup studies into North America vs “other” countries, as roughly 70% of studies were from North America. This latter comparison was limited to studies with > 80% laparoscopic approach to minimize possible confounding due to surgical approach. Since small samples have potentially lower sensitivity to capture VTE, sensitivity analysis was undertaken excluding the small studies (*n* < 2000 patients) to assess its influence on pooled incidence of the geographical subgroups.

### Heterogeneity

Heterogeneity was measured using Higgin’s *I*^2^, Cochrane’s Q, and Chi^2^. Given the nature of incidence data, high heterogeneity was expected due to large sample sizes and low variance. Therefore, thresholds for heterogeneity [21] were interpreted conservatively in line with recommendations regarding proportional meta-analysis [22].

### Publication Bias

We used funnel plots based on sample size (rather than standard error) as they have been shown to be a valid alternative for assessing publication bias in proportional meta-analysis [23], since traditional funnel plots may indicate asymmetry when no publication bias is present [22, 23]. In addition, recent guidelines recommend qualitative methods to appraise publication bias of incidence data [22]. Hence, we assessed publication bias using a combination of both.

## Results

### Search Results

**Fig. 1:**
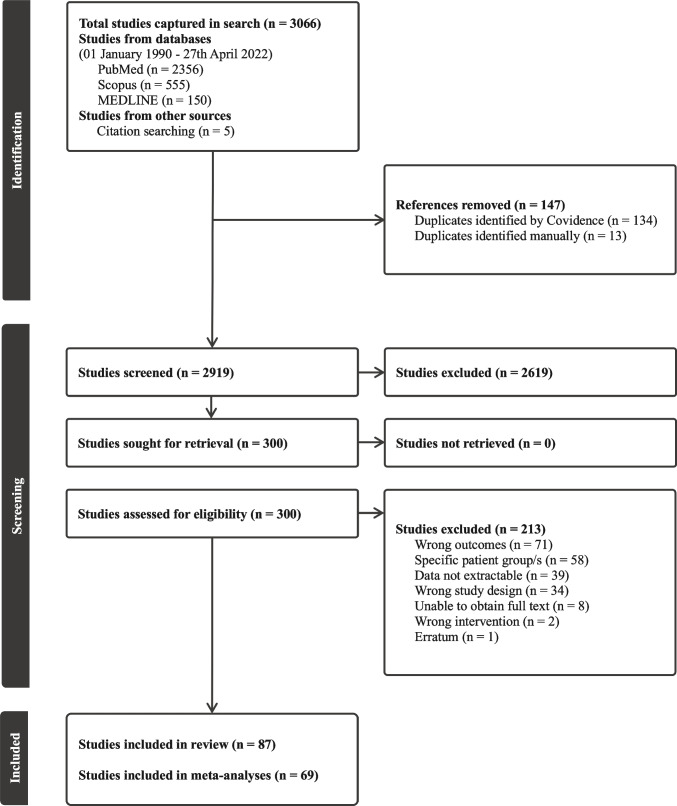
PRISMA flowchart of search and screening results

The PRISMA diagram (Fig. 1) shows that of 3066 retrieved articles, 87 were included in the review [5, 6, 9, 10, 14–17, 24–102], of which 68 were meta-analyzed. The studies excluded at full text and their reasons, as well as the included studies, and their subgroupings are available in Supplementary File 2.

### Study Characteristics

Table 1 outlines the studies included in the review (*N* = 4,991,683 patients). A total of 2,259,886 patients were subsequently excluded from meta-analyses due to data overlap or aggregated data. Data of the remaining 2,731,797 patients were meta-analyzed. The largest study included 540,959 patients [31] and the smallest comprised 39 patients [60].

**Table 1:** Characteristics of the 87 original studies included in the current review (4,991,683 patients)

Study	*N*	Country	Surgical procedure/s	Approach	Female (%)	Mean age *y* (range)	Mean BMI kg/m^2^	Study period
Almarshad 2020 [25]	374	Saudi Arabia	M	L 98.12% + O	72.99	36.07	**—**	2014–2018
Al-Mazrou 2021 [24]	7235	USA	BPD-DS	L 79.1% + R	73.17	43.79	51.0 ± 18.4	2015–2018
Arterburn 2014 [26]	7457	USA	RYGB, AGB	L	83.07	46 (45.8–46.3)	44.17	2005–2009
Bellen 2013 [10]	53	Brazil	**—**	O 73.6% + L	**—**	**—**	**—**	2007–2009
Biertho 2014 [26]	800	Canada	SG, BPD-DS	L	74	43.78	48 ± 7.57	2008–2011
Blackstone 2010 [27]	2416	USA	RYGB, AGB	L	77.28	45.4	47.5 ± 8.4	2001–2008
Celik 2014 [29]	2064	Netherlands	M	L > 95%	75	44.9	44.4 ± 6.2	2008–2011
Chan 2013 [14]	500	UK	M	L	75.4	44.7 (19–77)	49.2	2007–2010
Chung 2019 [30]	230,468	USA	M	L 90%	78.46	43.33	**—**	2000–2015
Clapp 2022 [31]	540,959	USA	RYGB, SG	L	80	44.4 (18–80)	5.2 ± 7.7	2017–2019
Clements 2009 [32]	956	USA	RYGB	L	82.76	41	49.1 ± 0.2	2000–2008
Cottam 2018 [33]	798	USA	RYGB, SIPS	**—**	73.06	45.65	48.98 ± 9.21	2010–2016
Cotter 2005 [34]	107	USA	RYGB	O 94.4% + L	78.5	40 (23–69)	51.3	2000–2001
Daigle 2018 [5]	135,413	USA	M	**—**	78.7	44.5 ± 12	45.7 ± 8.3	2015
Dang 2019 [6]	274,221	USA	RYGB, SG	L	79.2	44.6	45.51 ± 8.01	2015–2016
Doyon 2016 [35]	43,477	USA	RYGB	L	**—**	44.9	46.96 ± 8.21	2005–2012
ElChaar 2019 [36]	101,599	USA	RYGB, SG	L	**—**	**—**	**—**	2015
Eriksson 1997 [37]	328	Sweden	M	**—**^**a**^	77.13	38 (18–67)	44	1977–1993
Fanous 2012 [38]	711	USA	RYGB	L	80.17	45.15	51.67 ± 8.6	2007–2009
Fennern 2021 [39]	43,493	USA	RYGB, SG	L 94% + O	78	Md 45	**—**	2007–2015
Finks 2012 [40]	27,818	USA	M	L 95% + O	78	46	48	2006–2011
Flores 2022 [41]	788	Mexico	RYGB, SG	**—**	80.7	38.8	43.9 ± 7.8	2012/2018
Flum 2009 [42]	4610	USA	M	L 87.2% + O	78.9	44.5	**—**	2005–2007
Froehling 2013 [43]	396	USA	M	O ≥ 58% + L	81.6	43.8 (18–76)	Md 46.5	1987–2005
Gambhir 2020 [44]	369,032	USA	RYGB, SG	L	79.85	**—**	**—**	2015–2017
Gonzalez 2006 [45]	660	USA	RYGB	O 52.58% + L	**—**	**—**	**—**	1998–2004
Gorosabel Calzada 2022 [9]	675	Spain	M	L	67.26	43	45	2010–2015; 2019–2019
Greenstein 2012 [46]	5882	USA	M	L 91.43% + O	85.4	Md 47	Md 44	2005–2009
Guerrier 2018 [47]	114,362	USA	RYGB, SG	L (% **—**) + O	78.96	44.5	44.5	2010–2014
Hamad 2005 [48]	668	USA	M	O 84.9% + L	86	41.7	49.6 ± 8.4	2002–2002
Haskins 2015 [49]	102,869	USA	M	L 93.4% + O	78.925	45.11	46.08 ± 8.32	2005/2012
Haskins 2019 [50]	286,704	USA	RYGB, SG	L	79.66	44.93	45.26 ± 8.06	2015–2016
Hasley 2022 [51]	638	USA	SG	L	81.7	41.3	44.4	2010–2018
Helm 2017 [52]	59,424	USA	M	**—**	79.1	44.79	45.93 ± 8.16	2012–2014
Hess 1998 [53]	440	USA	BPD-DS	O	78.41	39	50	1988–^b^
Hu 2018 [54]	69,365	USA	M	L 90.04% + O	65.59	Md 45	**—**	2011–2014
Hussain 2020 [55]	212	Australia	M	L ≥ 52.83% + O	81.13	**—**	**—**	2013–2017
Imberti 2014 [56]	250	Italy	M	L ≥ 82.5% + O	79	40.9	44.4 ± 5.4	2004–2012
Inabnet 2010 [57]	3802	USA	M	L (% **—**) + O	**—**	44.22	48.51	2005–2007
Jamal 2015 [15]	4293	USA	M	L 99.615 + O	68.5	46.14 ± 9.81	48.45 ± 12.7	2005–2013
James 2021 [58]	153	USA	SG	L	81	**—**	47.9 ± 5.7	2017–2019
Kakarla 2011 [59]	28,634	USA	RYGB, GBn	L	**—**	**—**	**—**	2005–2008
Khoursheed 2013 [60]	39	Kuwait	RYGB	L	79.49	32.4	44.5 ± 6.1	2008–2010
Krell 2014 [61]	17,057	USA	RYGB	L	79	45.8	47.6 ± 7.89	2006–2012
Kruger 2014 [62]	3460	USA	M	L 99.83% + O	83.4	44 (18–74)	46.69 ± 6.58	2004–2013
Lech 2022 [63]	291	Poland	SG	L	79	40.3	45.3 ± 8.25	2016–2017
Leeman 2020 [64]	3319	Netherlands	RYGB, SG	**—**	82.46	40.41	43.27 ± 4.83	2014–2018
Li 2012 [65]	97,128	USA	RYGB, AGB	L 95% + O	78.87	46	45.5 ± 6.60	2007–2010
Lins 2015 [66]	209	Brazil	RYGB	L	**—**	40.2	41.5	2008–2013
Mabeza 2022 [67]	537,522	USA	RYGB, SG	L 97.3% + O	79.94	44.94	**—**	2016–2018
Magee 2010 [68]	735	UK	M	L	79.05	Md 42 (18–72)	Md 47.9	1997–2008
Masoomi 2011 [69]	304,515	USA	M	L 86% + O	79.9	44.1	**—**	2006–2008
McCullough 2006 [70]	109	USA	RYGB	L	75.2	46 (10.4)	48.7 ± 7.2	2001–2003
Miller 2004 [71]	255	USA	RYGB	L	82	43.2 (18–62)	50	2000–2003
Minhem 2018 [72]	21,131	USA	SG	L	78.21	44.35	46.07 ± 8.11	2010–2013
Modasi 2019 [73]	430,936	Canada	RYGB, SG	L	79.4	49.3	46.3 ± 7.94	2015–2017
Moussa 2021 [16]	4073	UK	**—**	**—**	79.6	Md 50 (IQR: 42–58)	Md 40.2	**—**
Nielsen 2018 [74]	59,041	USA	M	**—**	79	44.8 ± 11.8	45.9 ± 8.1	2012–2014
Nimeri 2013 [75]	29,990	USA/UAE	M	L 94.41% + O	69.8	36	47.4 ± 11	2009–2012
Nimeri 2018 [17]	66,845	USA/UAE	RYGB, SG	L	79.72	44.36 ± 11.91	47 ± 9.5	2010–2016
Nudel 2021 [76]	436,807	USA	RYGB, SG	L	79.3	44.7	45.4	2015–2017
Obeid 2007 [77]	2099	USA	RYGB, AGB	O 91.9% + L	84.85	44.65	54.4	2000–2006
Poulose 2005 [78]	69,072	USA	M	L (% **—**) + O	85	41.6 (41.2–42.0)	**—**	2002
Prasad 2012 [79]	108	India	SG	L	77.78	39.3 (15–62)	44.5 ± 6.8	2008–2011
Prystowsky 2005 [80]	106	USA	RYGB	L 75% + O	74	43 (26–67)	51	2004–2005
Quebbemann 2005 [81]	822	USA	M	L	**—**	43 (15–74)	45.2 ± 7.1	2000–2005
Raftopoulos 2008 [82]	308	USA	M	L 96.4% + O	82.79	43.35 (18–73)	46.95	2003–2007
Ramly 2017 [83]	66,078	USA	RYGB, RYGB/GBn removal	L	79.47	45.07 (16–90)	46.44 ± 8.28	2008–2014
Reames 2015 [84]	16,344	USA	RYGB	L	79	45.82	47.63 ± 7.83	2006–2012
Rezvani 2014 [85]	362	USA	BPD-DS	L	**—**	44.8	50 ± 7.1	2006–2012
Rezvani 2014 [86]	226	USA	BPD-DS	L	75.22	44.9	50.2	2009–2011
Rodríguez 2020 [87]	421	Chile	SG	L	65.56	35.35	35.94 ± 3.0791	2009–2019
Scholten 2002 [88]	481	USA	M	O 97.5% + L	83.16	44	51.05	1997–2000
Shah 2012 [89]	56	USA	AGB	L	**—**	38	50.9	2002–2007
Sharma 2020 [90]	737	USA	SG	L	56.31	45.3	43.9 ± 7.34	2012–2017
Singh 2012 [91]	170	USA	RYGB	**—**	46.47	43	47.8 ± 6.9	2004–2007
Spaniolas 2016 [92]	71,694	USA	M	L ≥ 89% + O	**—**	Md 45	Md 44.8	2006–2011
Steele 2011 [93]	17,434	USA	M	O 65.7% + L	82.01	43	**—**	2002–2005
Stroh 2012 [94]	11,835	Germany	M	**—**	72.5	42.2 (11–79)	48.8	2005–2010
Stroh 2016 [95]	29,561	Germany	M	L 98% + O	72.05	**—**	**—**	2005–2013
Surve 2022 [96]	5,017	USA	M	**—**	**—**	43.2 ± 12.1	44.6 ± 8.5	2016–2021
Thereaux 2018 [97]	110,824	France	M	L 95% + O	80.85	39.93 ± 11.6	**—**	2012–2014
Thereaux 2014 [98]	1008	France	RYGB	L	78.7	42.6 ± 11.6	47.6 ± 7.6	2004–2013
Westling 2002 [99]	116	Sweden	RYGB	O 74.1% + L	**—**	Md 35 (19–59)	Md 42	**—**^**b**^
Winegar 2011 [100]	73,921	USA	M	L 92.7% + O	79	45.8 ± 11.74	46 ± 7.85	2007–2009
Woo 2013 [101]	200	Korea	M	L	**—**	35 (14–63)	39	2009–2011
Young 2015 [102]	24,117	USA	RYGB, SG	L	78.09	44.8	46 ± 8.22	2010–2011

Due to space limitations, only the first author is cited

*BPD* biliopancreatic diversion, *D* duration, *DS* duodenal switch, *GB* gastric bypass, *GBn* gastric banding, *L* laparoscopic, *BMS* bariatric/metabolic surgery, *N* number of patients, *O* open approach, *RYGB* Roux-en-Y GB, *SG* sleeve gastrectomy, *y* years, *M* multiple, *Md* median, *R* robotic, *SIPS* Stomach Intestinal Pylorus-Sparing Surgery, — not explicitly reported/ cannot be computed

^a^Surgical approach not explicitly reported, assumed to be mostly open due to the sampling time frame being 1977–1993

^b^Time frame not explicitly reported, assumed to be before 2010 as publication date was before 2010

Geographically, 62 included studies (71.3%) were based on North American data, whereas 25 (28.7%) reported data from other countries. Surgical approach was > 80% laparoscopic in 60 studies (69.0%); > 50% open in 10 (11.5%); did not fulfill the above cut-offs for surgical approach in three (3.44%) [24, 55, 80]; and was not explicitly reported in 14 studies (16.1%). Thirty-two studies (36.8%) included data collected up to the end of 2010, 52 (59.8%) included data collected after 2010, and three studies (3.4%) did not report their data timeframe [16, 53, 99].

Thirty-six studies (41.4%) reported ≥ 3 MBS procedures, 15 (17.2%) undertook only RYGB, 14 (16.1%) included both RYGB and SG, seven (8.0%) undertook strictly SG, four (4.6%) conducted only BPD-DS, six (6.9%) included RYGB and adjustable gastric banding or removal, three (3.4%) reported other combinations [27, 33, 89], and two studies (2.3%) did not report their included procedures [10, 16].

Seventy-three studies (83.9%) reported the sex distribution of their sample, with females comprising a mean of 77.6%. Fourteen (16.1%) did not report sex distribution. Seventy-two studies (82.8%) reported the mean age of their sample, with an average of 42.9 ± 3.02 years, while seven (8%) provided the median age for the sample, and eight studies (9.2%) did not report age. Sixty-four studies (73.6%) reported mean BMI (46.1 ± 6.0 kg/m^2^ across all studies), six (6.9%) provided their median BMIs, while 17 studies (19.5%) did not report BMI.

Table 2 shows the time point/s of incidence provided by each included study (i.e., the specific meta-analysis/es that each study contributed to, as well as the studies that were excluded from primary (not sub-grouped) meta-analysis of any given time point due to significant overlap in data with other studies. Data sources of each included study are outlined in Supplementary File 3.

**Table 2:**
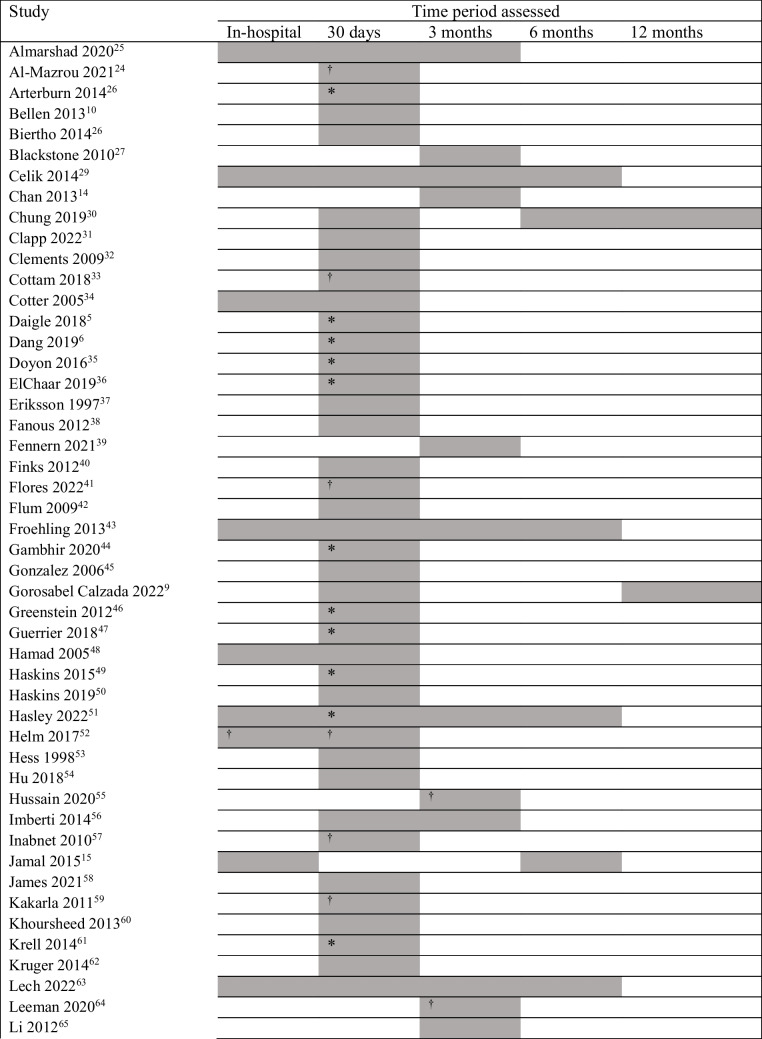

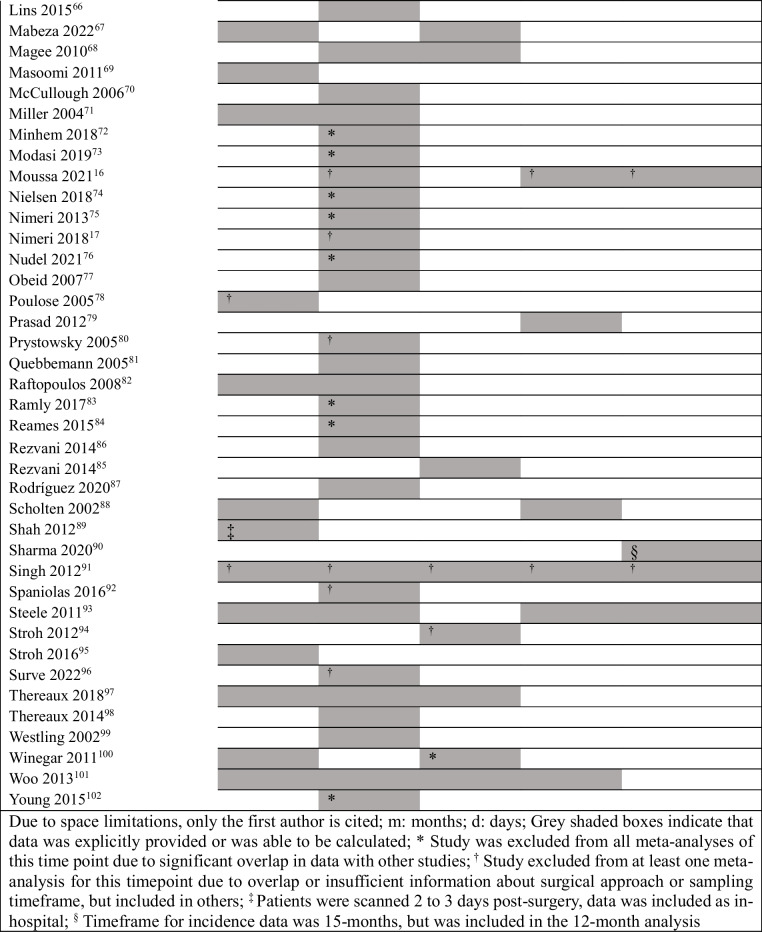
Timeframe of incidence provided by each included study

### Risk of Bias Appraisal

The mean risk of bias score was 5.82 ± 1.43, with a range of 3–8 (Supplementary File 4). Fifty studies (57.5%) scored six or higher, indicating a low risk of bias. The 20 studies with lower scores of 3–4 were mainly due to small sample sizes, potentially biased sampling frames, or poor reporting. Items 2 and 4 had the lowest number of studies receiving a score for them (50.57% and 42.53%, respectively). Average inter-rater agreement for the 10% of the studies randomly selected was 79.17% ± 17.25 across the nine items.

### Meta-analysis

The summary of findings of the meta-analyses at the different time points and their subgroupings is depicted in Table 3. Below, we detail the findings at each time point individually.

**Table 3:** Summary of findings

Timing of VTE		Studies	Total sample	Cases	Incidence	Heterogeneity		
		*N*	*N*	*N*	% of patients (95% CI)	*I*^2^ (%)	*Q*	Chi^2^, *p*
In-hospital								
Surgical approach	> 80% laparoscopic	14	1,064,822	1613	0.15 (0.13; 0.18)	72.00	47.23	0.00
	> 50% open	5	19,086	157	0.43 (0.05; 0.94)	75.00	16.03	0.00
Country	North America	8	921,508	1353	0.14 (0.11; 0.18)	80.00	34.31	0.00
	Other countries	6	143,404	260	0.18 (0.16; 0.20)	0.00	3.02	0.70
Year of study	Up to 2010 *	12	467,383	984	0.32 (0.18; 0.49)	96.00	301.16	0.00
	After 2010	10	745,191	1085	0.15 (0.12; 0.18)	61.00	22.93	0.00
30 Days								
Surgical approach	> 80%laparoscopic	30	1,380,293	7371	0.50 (0.33; 0.70)	99.00	4056.37	0.00
	> 50% open	10	22,295	466	2.02 (1.51; 2.57)	60.00	22.29	0.01
Country	North America	18	1,262,051	6960	0.58 (0.34; 0.87)	100.00	3985.54	0.00
	Other countries	12	117,090	402	0.34 (0.17; 0.55)	59.00	26.72	0.01
Year of study	Up to 2010 *	22	34,918	536	1.29 (0.81; 1.83)	91.00	228.18	0.00
	After 2010	25	1,421,225	7606	0.43 (0.27; 0.63)	99.00	4027.98	0.00
3 months								
Surgical approach	> 80%laparoscopic	14	796,797	4042	0.51 (0.38; 0.65)	95.00	242.97	0.00
	> 50% open	1	396	8	2.14 (0.83; 3.68)	N/A	N/A	N/A
Country	North America	6	681,559	3458	0.61 (0.40; 0.86)	98.00	230.26	0.00
	Other countries	7	113,174	574	0.37 (0.14; 0.65)	52.00	12.61	0.05
Year of study	Up to 2010 *	7	113,180	286	0.39 (0.19; 0.63)	87.00	44.47	0.00
	After 2010	11	697,485	3775	0.48 (0.39; 0.58)	82.00	54.81	0.00
6 months								
Surgical approach	> 80%laparoscopic	7	238,062	6177	0.72 (0.13; 1.52)	96.00	151.07	0.00
	> 50% open	3	18,311	537	2.36 (1.44; 3.41)	64.00	5.54	0.06
Country	North America	3	235,399	6165	1.45 (0.56; 2.69)	96.00	53.82	0.00
	Other countries	4	2663	12	0.31 (0.10; 0.74)	16.00	3.58	0.31
Year of study	Up to 2010 *	4	18,481	537	1.67 (0.57; 3.03)	83.00	17.64	0.00
	After 2010	7	238,062	6177	0.72 (0.13; 1.52)	96.00	151.07	0.00
12 months								
Surgical approach	> 80%laparoscopic	3	231,880	7559	0.78 (0.00; 3.49)	98.00	108.89	0.00
	> 50% open	1	16,929	579	3.38 (3.15; 3.70)	N/A	N/A	N/A
Country	North America	2	231,205	7559	1.60 (0.00; 5.00)	97.00	37.77	0.00
	Other countries	1	675	0	0.04 (0.00; 0.25)	N/A	N/A	N/A
Year of study	Up to 2010*	2	17,099	579	1.32 (0.00; 5.79)	93.00	14.75	0.00
	After 2010	3	231,880	7559	0.78 (0.00; 3.49)	98.00	108.89	0.00

*Includes 2010; *N/A* Not Applicable

### In-Hospital Incidence of VTE

**Fig. 2:**
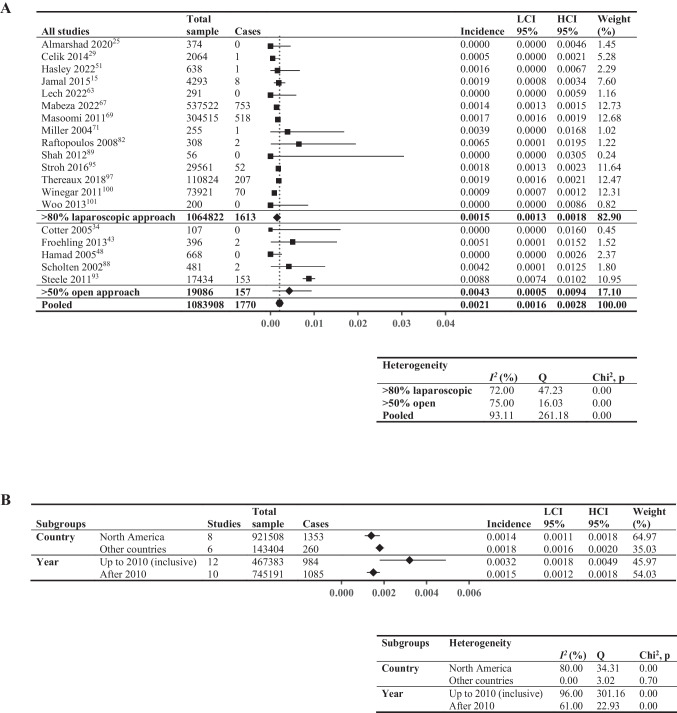
In-hospital incidence of venous thromboembolic events. Forest plot showing: **A** > 80% laparoscopic and > 50% open; **B** pooled results by two subgroupings—country (North America vs other countries, limited to studies comprising > 80% laparoscopic surgical approach to minimize confounding from surgical approach) and year (last year of data inclusion before and including 2010 vs after 2010, not limited by surgical approach). Square data points: incidence from individual studies; diamond-shaped data points: pooled values from subgroups; hexagonal data points: pooled values from all studies that reported relevant data

Meta-analysis of in-hospital incidence of VTE included 19 studies (1,083,908 patients, Fig. 2A), reporting a wide range of incidences (0–0.88%). Studies with > 80% laparoscopic approach exhibited lower pooled incidence of VTE (0.15%; *I*^2^ = 72%) compared to those with > 50% open approach (0.43%; *I*^2^ = 75%). Figure 2 B shows the subgroup analysis: North American studies had slightly lower incidence (0.14%,* I*^2^ = 80%) compared to other countries (0.18%; *I*^2^ = 0%), and studies using data collected up to the end of 2010 displayed higher incidence (0.32%; *I*^2^ = 96%) compared to those after 2010 (0.15%; *I*^2^ = 61%).

### Thirty-Day Cumulative Incidence of VTE

**Fig. 3:**
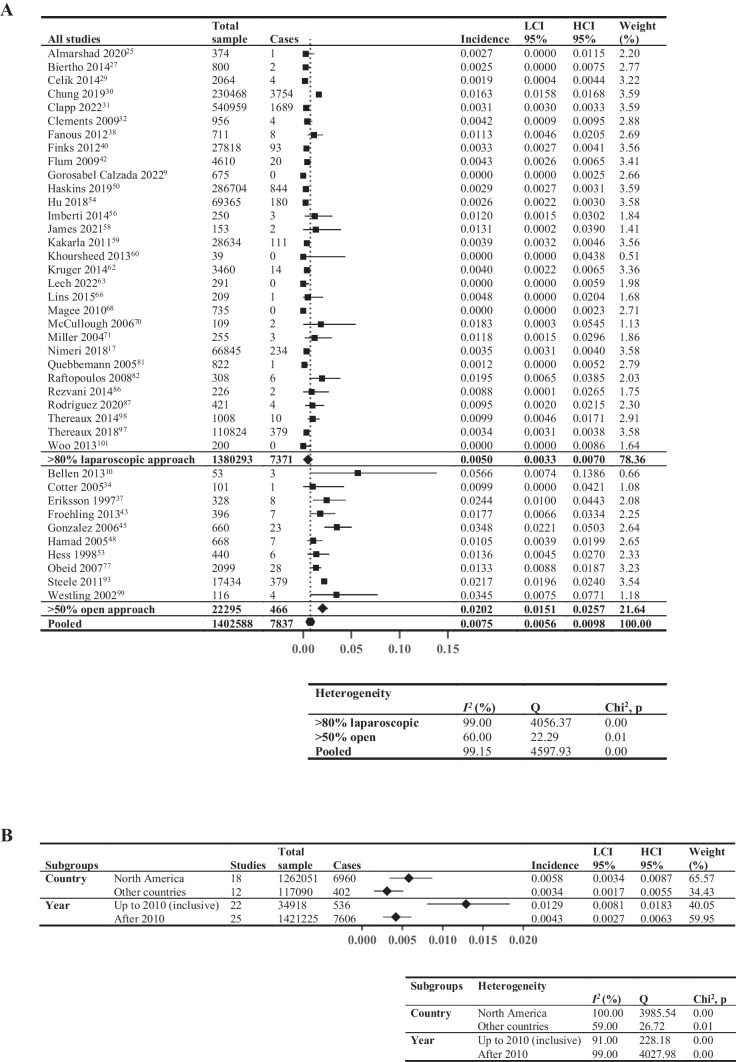
Thirty-day cumulative incidence of venous thromboembolic events. Forest plot showing: **A** > 80% laparoscopic and > 50% open; **B** pooled results by two subgroupings—country (North America vs other countries, limited to studies comprising > 80% laparoscopic surgical approach to minimize confounding from surgical approach) and year (last year of data inclusion before and including 2010 vs after 2010, not limited by surgical approach). Square data points: incidence from individual studies; diamond-shaped data points: pooled values from subgroups; hexagonal data points: pooled values from all studies that reported relevant data

Meta-analysis of 30-day cumulative incidence of VTE included 40 studies (1,402,588 patients, Fig. 3A), reporting a wide range of incidences (0–5.66%). Studies with > 80% laparoscopic approach exhibited lower incidence (0.50%; *I*^2^ = 99%) compared to those with > 50% open approach (2.02%; *I*^2^ = 60%). Figure 3 B shows the subgroup analysis: North American studies had higher incidence (0.58%;* I*^2^ = 100%) compared to other countries (0.34%; *I*^2^ = 59%), and studies with data collected up to the end of 2010 demonstrated higher incidence (1.29%, *I*^2^ = 91%) compared to those after 2010 (0.43%; *I*^2^ = 99%).

### Three-Month Cumulative Incidence of VTE

**Fig. 4:**
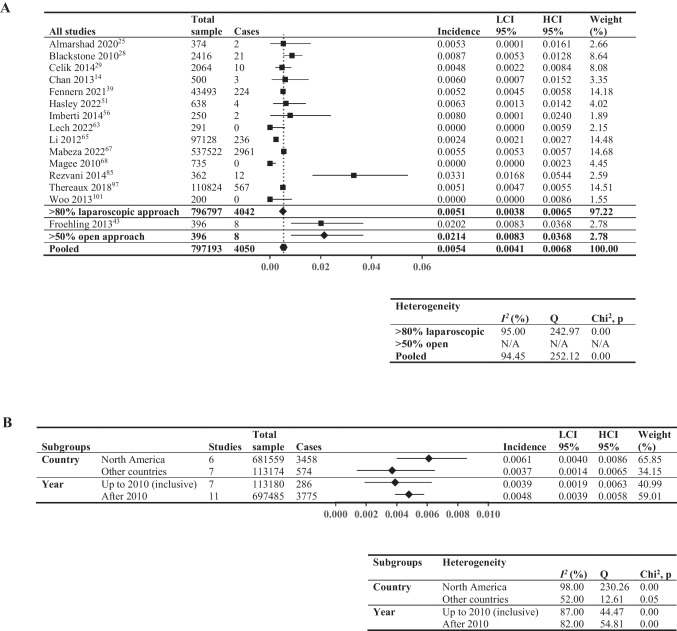
Three-month cumulative incidence of venous thromboembolic events. Forest plot showing: **A** > 80% laparoscopic and > 50% open; **B** pooled results by two subgroupings—country (North America vs other countries, limited to studies comprising > 80% laparoscopic surgical approach to minimize confounding from surgical approach) and year (last year of data inclusion before and including 2010 vs after 2010, not limited by surgical approach). Square data points: incidence from individual studies; diamond-shaped data points: pooled values from subgroups; hexagonal data points: pooled values from all studies that reported relevant data

Meta-analysis of the 3-month cumulative incidence of VTE included 15 studies (797,193 patients, Fig. 4A), reporting a wide range of incidences (0%-3.31%). Studies with > 80% laparoscopic approach exhibited lower incidence (0.51%; *I*^2^ = 95%) compared to those with > 50% open approach (2.14%; *I*^2^ not applicable); Fig. 4B shows the subgroup analysis: North American studies had higher incidence (0.61%;* I*^2^ = 98%) compared to other countries (0.37%; *I*^2^ = 52%); and studies using data up to the end of 2010 had lower incidence (0.39%; *I*^2^ = 87%) compared to those after 2010 (0.48%; *I*^2^ = 82%).

### Six-Month Cumulative Incidence of VTE

**Fig. 5:**
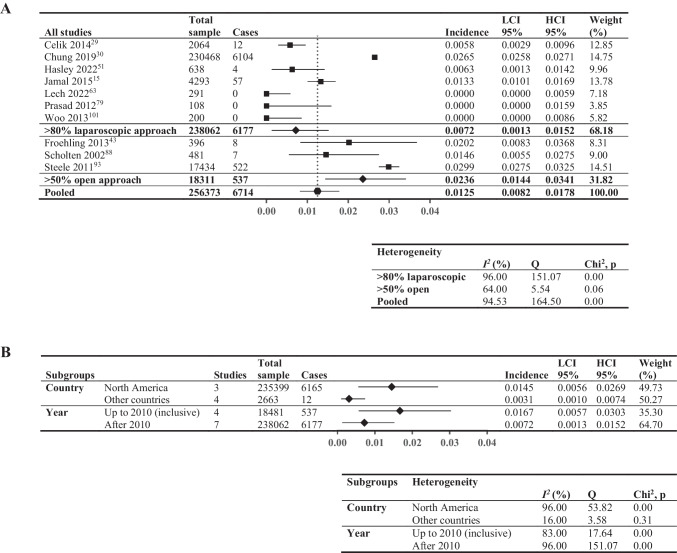
Six-month cumulative incidence of venous thromboembolic events. Forest plot showing: **A** > 80% laparoscopic and > 50% open; **B** pooled results by two subgroupings—country (North America vs other countries, limited to studies comprising > 80% laparoscopic surgical approach to minimize confounding from surgical approach) and year (last year of data inclusion before and including 2010 vs after 2010, not limited by surgical approach). Square data points: incidence from individual studies; diamond-shaped data points: pooled values from subgroups; hexagonal data points: pooled values from all studies that reported relevant data

Meta-analysis of the 6-month cumulative incidence of VTE included 10 studies (256,373 patients, Fig. 5A), reporting a wide range of incidences (0–2.99%). Studies with > 80% laparoscopic approach exhibited lower incidence (0.72%; *I*^2^ = 96%) compared to those with > 50% open approach (2.36%; *I*^2^ = 64%). Figure 5 B shows the subgroup analysis: North American studies had higher incidence (1.45%;* I*^2^ = 96%) compared to other countries (0.31%; *I*^2^ = 16%), and studies using data up to the end of 2010 displayed higher incidence (1.67%; *I*^2^ = 83%) in comparison with those after 2010 (0.72%; *I*^2^ = 96%).

### Twelve-Month Cumulative Incidence of VTE

**Fig. 6:**
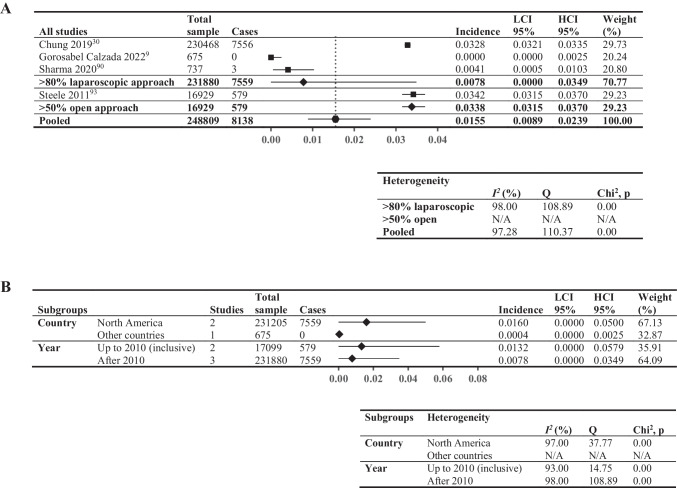
Twelve-month cumulative incidence of venous thromboembolic events. Forest plot showing: **A** > 80% laparoscopic and > 50% open; **B** pooled results by two subgroupings—country (North America vs other countries, limited to studies comprising > 80% laparoscopic surgical approach to minimize confounding from surgical approach) and year (last year of data inclusion before and including 2010 vs after 2010, not limited by surgical approach). Square data points: incidence from individual studies; diamond-shaped data points: pooled values from subgroups; hexagonal data points: pooled values from all studies that reported relevant data

Meta-analysis of the 12-month cumulative incidence of VTE included six studies (248,809 patients, Fig. 6A). Included studies reported a wide range (0–3.42%) of incidences. Studies with > 80% laparoscopic approach exhibited lower incidence (0.78%; *I*^2^ = 98%) compared to those with > 50% open approach (3.38%; *I*^2^ not applicable). Figure 6 B shows the subgroup analysis: North American studies had higher incidence (1.60%;* I*^2^ = 97%) compared to other countries (0.04%; *I*^2^ not applicable), and studies using data up to the end of 2010 displayed higher incidence (1.32%; *I*^2^ = 93%) in comparison with those after 2010 (0.78%; *I*^2^ = 98%).

### Incidence of VTE Within 30 Days: In-hospital vs Post-Discharge

Meta-analysis of 11 studies that reported both in-hospital and 30-day incidence (Supplementary File 5) showed that 60% (95% CI 57–63%; *I*^2^ = 88.16%) of the 30-day VTE occurred after discharge, based on 1073 events.

### VTE Over Time

**Fig. 7:**
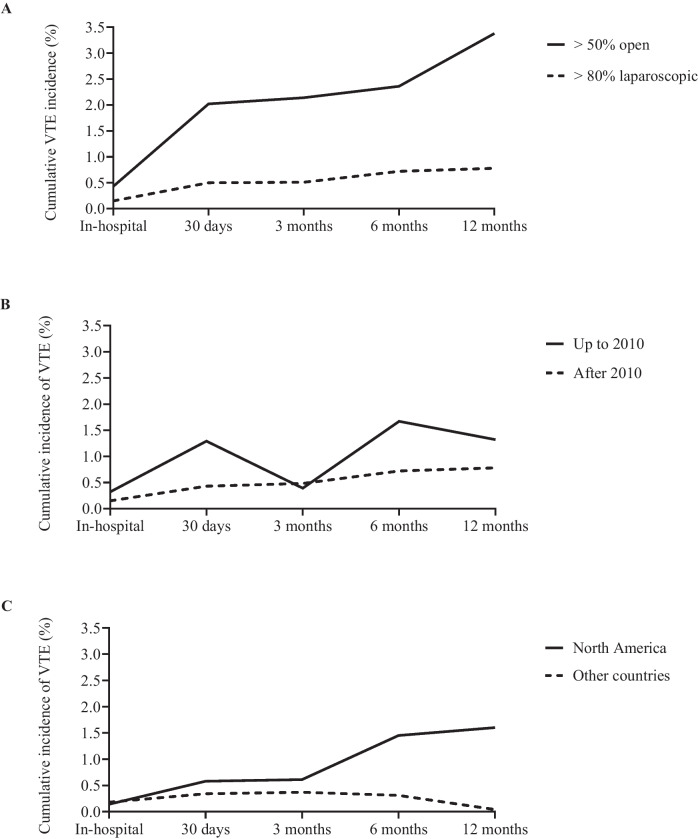
Total and sub-grouped cumulative incidence of VTE after metabolic and bariatric surgery across time: **A** by surgical approach (> 50% open vs > 80% laparoscopic), **B** by study age (up to and including 2010 vs after 2010), **C** by geographical origin (North America vs other countries). VTE, venous thromboembolic events

Cumulative incidence of VTE over time is depicted in Fig. 7. Incidence generally increased up to the last timepoint examined (12 months post-MBS). Incidence for > 80% laparoscopic approach was consistently lower compared to the > 50% open approach (Fig. 7A). Subgroup analyses displayed variations across time; incidence from North American studies was higher for most timepoints (based on > 80% laparoscopic procedures only) (Fig. 7C). Sensitivity analysis removing studies with sample sizes < 2000 patients increased the incidence for both subgroups and largely accounted for differences at 30 days (North America 0.43% vs other 0.31%) and 3 months (North America 0.49% vs other 0.51%), but not at 6 months (North America 1.83% vs other 0.48%). Sensitivity analysis was not possible for 12-month data.

### Publication Bias

**Fig. 8:**
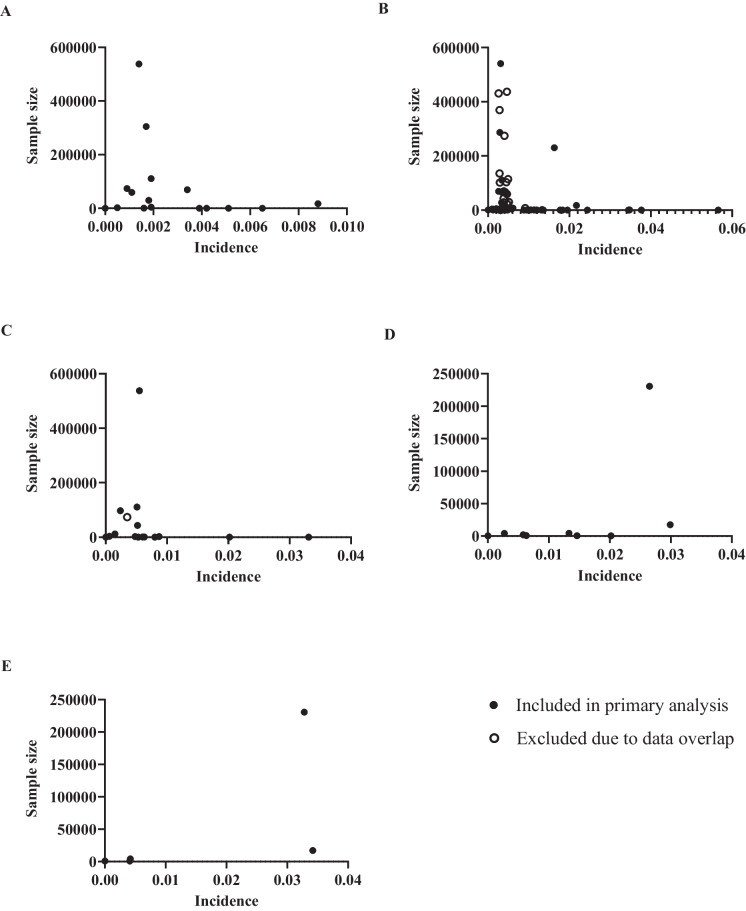
Panel of Funnel plots of all included studies presenting data for cumulative incidence of venous thromboembolic events: **A** in-hospital, **B** 30-day, **C** 3-month, **D** 6-month, and **E** 12-month. Solid black dots indicate studies included in the primary meta-analysis; open circles indicate studies excluded from the primary meta-analysis due to significant overlap of included patient data

Figure 8 depicts the funnel plots of cumulative incidence of VTE. At some timepoints, more studies reported lower incidence (Fig. 8A–C), and there was a relative paucity of studies of moderate sample sizes; hence, studies clustered at the upper (larger sample sizes) and lower (smaller sample sizes) ends of the *Y* axis (Figs. 8C–E). Qualitatively, countries outside of North America were underrepresented. Roughly three quarters of the studies reported North American data, with many using data registries. For instance, 28 (40.58%) of 69 studies reporting 30-day incidence used North American registry data, introducing considerable overlap of patient data across studies. Figure 8 B shows an unusual ‘stacking’ pattern of very similar incidences of VTE suggesting the duplicate use of patient data by different studies.

## Discussion

Patients with obesity are at risk of VTE in the post-MBS period [8, 100], and those who develop VTE have an increased risk of mortality [5, 6]. Despite this, no previous study has meta-analyzed the incidence of VTE after MBS. The present systematic review and meta-analysis presented high-quality cumulative incidences of VTE pooled from nearly 5 million patients worldwide. The in-hospital, 30-day, and 3-, 6- and 12-month incidences provide clinically relevant and meaningful information regarding the timing and patterns of VTE, to guide the follow-up, detection, and prevention of this life-threatening complication. The review also explored the influence of surgical approach, geographical origin, and study age on incidence of VTE. To our knowledge, this is the first study to undertake such a task.

In terms of the incidence of VTE at the five timepoints under examination, our observed cumulative incidence of VTE exhibited an increasing trend in-hospital, and at 30 days and 3, 6 and 12 months, for the > 80% laparoscopic approach (0.15%, 0.50%, 0.51%, 0.72%, and 0.78% respectively) and for the > 50% open approach (0.43%, 2.02%, 2.14%, 2.36%, and 3.38% respectively). Such increasing pattern is consistent with two studies that reported incidences of VTE after MBS of 0.88%_in-hospital_, 2.17%_1 month_, and 2.99%_6 month_ [93] and 0.3%_7 days_, 1.9%_30 days_, 2.1%_3 months_, and 2.1%_6 months_ [43]. Therefore, MBS patients require clinical vigilance to continue for an extended period, in order to identify VTE and reduce the risk of morbidity and mortality.

Individual studies reported a wide range of incidences at each timepoint. Such variations could be due to patient features such as age, BMI, or comorbidity [103]; surgical characteristics, such as operative time [103], MBS procedure, or surgical approach [100, 104, 105]; or study characteristics, such as study design, years of data acquisition, and sample size.

Across studies that reported both in-hospital and 30-day VTE, 60% of events occurred after discharge, concurring with previous reports where up to 80% of VTE occurred after discharge [52, 93, 100]. Higher post-discharge incidence of VTE might be partly attributed to short in-hospital stays of only a few days [106, 107], compared to longer post-discharge periods. Similarly, we found that most VTE occurred within the first 30 days, consistent with observations from some of the included studies [43, 52]. This further highlights the importance of vigilance during this period.

The present study noted that cumulative incidence across the > 80% laparoscopic studies was consistently lower than the > 50% open approach for all timepoints, consistent with previous findings at 30 days [105], 90 days [100], and 5 years [104]. Notwithstanding, some literature has demonstrated no differences in VTE outcomes between laparoscopic vs open approaches [108–110].

As for the subgroup analyses, we explored the effects of study age and geographical origin.

Studies using data up to the end of 2010 demonstrated higher incidences at most timepoints, compared to more recent studies, likely due to the larger proportion of > 50% open approach studies in the former subgroup. Factors that have contributed to the reduction in VTE since the turn of the century include the shift from open to laparoscopic approaches, MBS technical advancements, pre-/post-surgery thromboprophylaxis, and enhanced recovery regimens [68, 111–115].

To explore geographical differences, we compared North American studies to other countries. Despite limiting this to > 80% laparoscopic studies to minimize confounding from surgical approach, incidence from North American studies was higher for most timepoints. Sensitivity analysis removing studies with less than 2000 patients increased the incidence of the other countries group closer to that of North American studies at 30 days and 3 months, the timepoints where both subgroups used large samples from registry data, indicating the influence of sample size.

The current review identified only one study that assessed outcomes beyond the first few years after MBS [16]. This study found that over a median of 10.7 years post-surgery, MBS patients exhibited significantly less VTE compared to non-MBS patients matched for sex, age, and baseline BMI [16]. This suggests that despite our observed shorter-term incidence of VTE, MBS appears to offer “protection” (e.g., decreases in BMI), resulting in lower long-term risk of VTE [7]. Future research should include longer-term assessment of VTE after MBS.

Collectively, the above suggests that a deeper understanding of the variations in VTE across time must consider the interrelationships between surgical approach (and hence study age) and sample size (and hence the use of data registries and geographical origin), amongst other factors.

In terms of the quality of estimates, risk of bias within and across studies and heterogeneity, slightly more than half of the included studies exhibited low risk of bias. Some of the studies displaying higher risk of bias were due to small sample sizes, potentially biased sampling frames, or poor reporting. North American studies were over-represented, with many utilizing large national/regional registries. This led to considerable overlap of patient data, which increased our efforts to identify and exclude overlapping data to ensure the validity of the meta-analysis. Heterogeneity in the overall meta-analyses was high at all timepoints. Subgrouping reduced some heterogeneity; however, it remained high for the > 80% laparoscopic approach and the North American subgroups, both of which included the studies with the largest sample sizes and lowest variance. This is consistent with others who noted that measures of heterogeneity such as Higgin’s *I*^2^ may indicate high heterogeneity in proportional meta-analysis, even when data are consistent [22].

This review has some limitations. Many studies reported 30-day incidence, while others reported inconsistent timepoints, rendering interpretations of incidence across individual studies difficult. However, this variation enabled us to assess cumulative incidence and its patterns over time. Additionally, as most studies were retrospective, based on patient charts/records, pooled incidences are likely to reflect *symptomatic* VTE. As it was only possible to use the > 80% laparoscopic and > 50% open subgroups, this would have resulted in some contamination within the subgroups, suggesting that our observed VTE differences between surgical approaches could be underestimated. It would have been beneficial to include elements of the prophylaxis undertaken as well as operative time in the analysis. However, the extent of non-reporting, aggregated or undetailed reporting of these items, and in the case of prophylaxis, the numerous and wide variations in the chemo/mechanical prophylaxis protocols used singly or in combination at different times and durations of administration, with or without inferior vena cava filters, transfusions, or stoppage of chemical thromboprophylaxis where required would result in countless combinations thereof, which mitigated against a meaningful analysis. Notwithstanding, some of the included studies reported that duration of surgery for patients who experienced VTE after MBS was longer than that of matched control patients [29], and that operative time was significantly longer in patients who experienced a post-operative VTE [52] and a significant predictor of or associated with of post-operative VTE [40, 44].

Future studies would benefit from prospective designs, better (non-aggregated) reporting of sample/procedure characteristics and timeframes, assessment of longer-term VTE, and greater representation from outside of North America. Future meta-analyses should be aware of studies utilizing large national/regional registries that could lead to considerable overlap of patient data. Future researchers should be mindful of the differences across data registries when conducting research to ensure that significant proportions of events are not missed. The current study clearly demonstrated that the time course of VTE post-surgery is dynamic. As such, researchers presenting primary research on such complications need to clearly relate reported incidences to a given timeframe post-surgery, and those synthesizing such studies should be careful not to aggregate incidences related to different timeframes, since this would render any reported values meaningless.

This study has many strengths. We assessed the pooled incidence of VTE after MBS at five timepoints. Subgroup analysis included surgical approach, geographical origin of the studies, and study age. We meticulously identified potential overlap of patient data, including that from large registries, and excluded such studies from the meta-analysis, enhancing the internal validity [116]. The extremely large number of patients worldwide enhances the external validity and generalizability of the findings. To our knowledge, this is the most extensive and comprehensive systematic review/meta-analysis of VTE after MBS over several timepoints that has been undertaken, and probably the largest systematic review/meta-analysis conducted to date in the field of surgery/health in general in terms of the number of patients.

## Conclusion

We pooled a large number of studies and patients worldwide to provide high-quality estimates of VTE and valuable insights into its patterns over time. For studies that utilized a mainly laparoscopic approach, in-hospital incidence of VTE and cumulative incidence at 30 days and 3, 6 and 12 months were 0.15%, 0.50%, 0.51%, 0.72%, and 0.78% respectively. Most VTE occurred in the first 30 days, of which 60% was after discharge, although we observed some VTE up to our last timepoint. Incidence was consistently lower for laparoscopic compared to open MBS. Lower incidences from studies outside of North America were largely due to smaller sample sizes. Deeper understanding of the variations in VTE across time must consider the interrelationships between surgical approach, geographical origin, study age, and sample size, amongst other factors. Post-operative surveillance needs to be particularly vigilant after discharge and continue thereafter for an extended period to detect VTE and reduce the risk of associated morbidity and mortality. These findings provide clinically relevant estimates of VTE to inform policy, clinical practice, and research.

## References

[1] Elgenaied I, El Ansari W, Elsherif MA (2020). Factors associated with complete and partial remission, improvement, or unchanged diabetes status of obese adults 1 year after sleeve gastrectomy. Surg Obes Relat Dis.

[2] Elhag W, El Ansari W, Abdulrazzaq S (2018). Evolution of 29 anthropometric, nutritional, and cardiometabolic parameters among morbidly obese adolescents 2 years post sleeve gastrectomy. Obes Surg.

[3] Chao GF, Montgomery JR, Abou Azar S (2021). Venous thromboembolism: risk factors in the sleeve gastrectomy era. Surg Obes Relat Dis.

[4] Khorgami Z, Shoar S, Andalib A (2017). Trends in utilization of bariatric surgery, 2010–2014: sleeve gastrectomy dominates. Surg Obes Relat Dis.

[5] Daigle CR, Brethauer SA, Tu C (2018). Which postoperative complications matter most after bariatric surgery? Prioritizing quality improvement efforts to improve national outcomes. Surg Obes Relat Dis.

[6] Dang JT, Switzer N, Delisle M (2019). Predicting venous thromboembolism following laparoscopic bariatric surgery: development of the BariClot tool using the MBSAQIP database. Surg Endosc.

[7] El Ansari W, El-Ansari K (2020). Missing something? A scoping review of venous thromboembolic events and their associations with bariatric surgery. Refining the evidence base. Ann Med Surg.

[8] El Ansari W, Sathian B, El-Menyar A (2020). Venous thromboembolic events after bariatric surgery: protocol for a systematic review and meta-analysis. Int J Surg Protoc.

[9] Gorosabel Calzada M, Hernández Matías A, Andonaegui de la Madriz A (2022). Thrombotic and hemorrhagic risk in bariatric surgery with multimodal rehabilitation programs comparing 2 reduced guidelines for pharmacological prophylaxis. Cirugia Espanola.

[10] Bv Bellen, Godoy IdB, Reis AA (2013). Venous insufficiency and thromboembolic disease in bariatric surgery patients. Arq Gastroenterol.

[11] Uhe I, Douissard J, Podetta M (2022). Roux-en-Y gastric bypass, sleeve gastrectomy, or one-anastomosis gastric bypass? A systematic review and meta-analysis of randomized-controlled trials. Obesity.

[12] Verhoeff K, Mocanu V, Zalasky A (2022). Evaluation of metabolic outcomes following SADI-S: a systematic review and meta-analysis. Obes Surg.

[13] Boppre G, Diniz-Sousa F, Veras L (2022). Can exercise promote additional benefits on body composition in patients with obesity after bariatric surgery? A systematic review and meta-analysis of randomized controlled trials. Obes Sci Pract.

[14] Chan MM, Hamza N, Ammori BJ (2013). Duration of surgery independently influences risk of venous thromboembolism after laparoscopic bariatric surgery. Surg Obes Relat Dis.

[15] Jamal MH, Corcelles R, Shimizu H (2015). Thromboembolic events in bariatric surgery: a large multi-institutional referral center experience. Surg Endosc.

[16] Moussa O, Ardissino M, Tang A (2021). Long-term impact of bariatric surgery on venous thromboembolic risk: a matched cohort study. Ann Surg.

[17] Nimeri AA, Bautista J, Ibrahim M (2018). Mandatory risk assessment reduces venous thromboembolism in bariatric surgery patients. Obes Surg.

[18] Page MJ, McKenzie JE, Bossuyt PM (2021). The PRISMA 2020 statement: an updated guideline for reporting systematic reviews. Int J Surg.

[19] Loney PL, Chambers LW, Bennett KJ (1998). Critical appraisal of the health research literature: prevalence or incidence of a health problem. Chronic Dis Can.

[20] Barendregt JJ, Doi SA, Lee YY (2013). Meta-analysis of prevalence. J Epidemiol Community Health.

[21] Higgins JPT, Thompson SG, Deeks JJ (2003). Measuring inconsistency in meta-analyses. BMJ.

[22] Barker TH, Migliavaca CB, Stein C (2021). Conducting proportional meta-analysis in different types of systematic reviews: a guide for synthesisers of evidence. BMC Med Res Methodol.

[23] Hunter JP, Saratzis A, Sutton AJ (2014). In meta-analyses of proportion studies, funnel plots were found to be an inaccurate method of assessing publication bias. J Clin Epidemiol.

[24] Al-Mazrou AM, Cruz MV, Dakin G (2021). Robotic duodenal switch is associated with outcomes comparable to those of laparoscopic approach. Obes Surg.

[25] Almarshad FM, Almegren M, Alshuaibi T (2020). Thromboprophylaxis after bariatric surgery. Blood Res.

[26] Arterburn D, Powers JD, Toh S (2014). Comparative effectiveness of laparoscopic adjustable gastric banding vs laparoscopic gastric bypass. JAMA Surg.

[27] Biertho L, Lebel S, Marceau S (2014). Laparoscopic sleeve gastrectomy: with or without duodenal switch? A consecutive series of 800 cases. Dig Surg.

[28] Blackstone RP, Cortés MC (2010). Metabolic acuity score: effect on major complications after bariatric surgery. Surg Obes Relat Dis.

[29] Celik F, Bounif F, Fliers JM (2014). The impact of surgical complications as a main risk factor for venous thromboembolism: a multicenter study. Obes Surg.

[30] Chung AY, Strassle PD, Schlottmann F (2019). Trends in utilization and relative complication rates of bariatric procedures. J Gastrointest Surg.

[31] Clapp B, Grasso S, Gamez J (2022). Does accreditation matter? An analysis of complications of bariatric cases using the Metabolic and Bariatric Surgery Accreditation and Quality Improvement Program and National Quality Improvement Program databases. Surg Obes Relat Dis.

[32] Clements RH, Yellumahanthi K, Ballem N (2009). Pharmacologic prophylaxis against venous thromboembolic complications is not mandatory for all laparoscopic Roux-en-Y gastric bypass procedures. J Am Coll Surg..

[33] Cottam A, Cottam D, Zaveri H (2018). An analysis of mid-term complications, weight loss, and type 2 diabetes resolution of Stomach Intestinal Pylorus Sparing surgery (SIPS) versus Roux-En-Y gastric bypass (RYGB) with three-year follow-up. Obes Surg.

[34] Cotter SAP, Cantrell WPB, Fisher BMDF (2005). Efficacy of venous thromboembolism prophylaxis in morbidly obese patients undergoing gastric bypass surgery. Obes Surg.

[35] Doyon L, Moreno-koehler A, Ricciardi R (2016). Resident participation in laparoscopic Roux-en-Y gastric bypass: a comparison of outcomes from the ACS-NSQIP database. Surg Endosc.

[36] El Chaar M, Stoltzfus J, Gersin K (2019). A novel risk prediction model for 30-day severe adverse events and readmissions following bariatric surgery based on the MBSAQIP database. Surg Obes Relat Dis.

[37] Eriksson SMDP, Backman LMDP, Ljungström KG (1997). The incidence of clinical postoperative thrombosis after gastric surgery for obesity during 16 years. Obes Surg..

[38] Fanous M, Carlin A (2012). Surgical resident participation in laparoscopic Roux-en-Y bypass: is it safe?. Surgery.

[39] Fennern EB, Farjah F, Chen JY (2021). Use of post-discharge heparin prophylaxis and the risk of venous thromboembolism and bleeding following bariatric surgery. Surg Endosc.

[40] Finks JF, English WJ, Carlin AM, et al (2012) Predicting risk for venous thromboembolism with bariatric surgery: results from the Michigan Bariatric Surgery Collaborative. Ann Surg 255(6). 10.1097/SLA.0b013e31825659d4 22566018

[41] Flores JE, Berrones R, Guilbert L (2022). Complications rate variability after bariatric surgery and the importance of standardization of a reporting system. J Gastrointest Surg.

[42] Flum DR, Belle SH, King WC (2009). Perioperative safety in the longitudinal assessment of bariatric surgery. N Engl J Med.

[43] Froehling DA, Daniels PR, Mauck KF (2013). Incidence of venous thromboembolism after bariatric surgery: a population-based cohort study. Obes Surg.

[44] Gambhir S, Inaba CS, Alizadeh RF (2020). Venous thromboembolism risk for the contemporary bariatric surgeon. Surg Endosc.

[45] Gonzalez R, Haines K, Nelson LG (2006). Predictive factors of thromboembolic events in patients undergoing Roux-en-Y gastric bypass. Surg Obes Relat Dis..

[46] Greenstein AJ, Wahed AS, Adeniji A (2012). Prevalence of adverse intraoperative events during obesity surgery and their sequelae. J Am Coll Surg.

[47] Guerrier JB, Dietch ZC, Schirmer BD (2018). Laparoscopic sleeve gastrectomy is associated with lower 30-day morbidity versus laparoscopic gastric bypass: an analysis of the American College of Surgeons NSQIP. Obes Surg.

[48] Hamad GG, Choban PS (2005). Enoxaparin for thromboprophylaxis in morbidly obese patients undergoing bariatric surgery: findings of the prophylaxis against VTE outcomes in Bariatric Surgery Patients Receiving Enoxaparin (PROBE) Study. Obes Surg.

[49] Haskins IN, Amdur R, Sarani B (2015). Congestive heart failure is a risk factor for venous thromboembolism in bariatric surgery. Surg Obes Relat Dis.

[50] Haskins IN, Rivas L, Ju T (2019). The association of IVC filter placement with the incidence of postoperative pulmonary embolism following laparoscopic bariatric surgery: an analysis of the Metabolic and Bariatric Surgery Accreditation and Quality Improvement Project. Surg Obes Relat Dis.

[51] Hasley RB, Aly S, Carter CO (2022). Application of the Caprini risk assessment model to select patients for extended thromboembolism prophylaxis after sleeve gastrectomy. J Gastrointest Surg.

[52] Helm MC, Simon K, Higgins R (2017). Perioperative complications increase the risk of venous thromboembolism following bariatric surgery. Am J Surg.

[53] Hess DS, Hess DW (1998). Biliopancreatic diversion with a duodenal switch. Obes Surg.

[54] Hu Q, Shi L, Chen L (2018). Seasonality in the adverse outcomes in weight loss surgeries. Surg Obes Relat Dis.

[55] Hussain Z, Peterson GM, Mirkazemi C (2020). Risk of venous thromboembolism, use of enoxaparin and clinical outcomes in obese patients undergoing laparoscopic adjustable gastric band surgery: a retrospective study. Medicine (Baltimore).

[56] Imberti D, Baldini E, Pierfranceschi MG (2014). Prophylaxis of venous thromboembolism with low molecular weight heparin in bariatric surgery: a prospective, randomised pilot study evaluating two doses of Parnaparin (BAFLUX Study). Obes Surg.

[57] Inabnet WB, Belle SH, Bessler M (2010). Comparison of 30-day outcomes after non-LapBand primary and revisional bariatric surgical procedures from the Longitudinal Assessment of Bariatric Surgery study. Surg Obes Relat Dis.

[58] James TJ, Sener SF, Nguyen JD (2021). Introducing a bariatric surgery program at a large urban safety net medical center serving a primarily Hispanic patient population. Obes Surg.

[59] Kakarla VR, Nandipati K, Lalla M (2011). Are laparoscopic bariatric procedures safe in superobese (BMI ≥50 kg/m2) patients? An NSQIP data analysis. Surg Obes Relat Dis.

[60] Khoursheed M, Al-Bader I, Al-Asfar F (2013). Therapeutic effect of low-molecular weight heparin and incidence of lower limb deep venous thrombosis and pulmonary embolism after laparoscopic bariatric surgery. Surg Laparosc Endosc Percutan Tech.

[61] Krell RW, Birkmeyer NJO, Reames BN (2014). Effects of resident involvement on complication rates after laparoscopic gastric bypass. J Am Coll Surg.

[62] Kruger RS, Pricolo VE, Streeter TT (2014). A bariatric surgery center of excellence: operative trends and long-term outcomes. J Am Coll Surg.

[63] Lech P, Michalik M, Waczyński K (2022). Effectiveness of prophylactic doses of tranexamic acid in reducing hemorrhagic events in sleeve gastrectomy. Langenbecks Arch Surg.

[64] Leeman M, Biter LU, Apers JA (2020). A single-center comparison of extended and estricted thromboprophylaxis with LMWH after metabolic surgery. Obes Surg.

[65] Li W, Gorecki P, Semaan E (2012). Concurrent prophylactic placement of inferior vena cava filter in gastric bypass and adjustable banding operations in the Bariatric Outcomes Longitudinal Database. J Vasc Surg.

[66] Lins DC, Campos JM, de Paula PS (2015). C-reactive protein in diabetic patients before gastric bypass as a possible marker for postoperative complication. Arq Bras Cir Dig..

[67] Mabeza RM, Lee C, Verma A, et al. Factors and outcomes associated with venous thromboembolism following bariatric surgery. Am Surg*.* 2022:31348221103645. 10.1177/00031348221103645 35611767

[68] Magee CJ, Barry J, Javed S (2010). Extended thromboprophylaxis reduces incidence of postoperative venous thromboembolism in laparoscopic bariatric surgery. Surg Obes Relat Dis.

[69] Masoomi HMD, Buchberg BMD, Reavis KMMD (2011). Factors predictive of venous thromboembolism in bariatric surgery. Am Surg.

[70] McCullough PA, Gallagher MJ, deJong AT (2006). Cardiorespiratory fitness and short-term complications after bariatric surgery. Chest.

[71] Miller MT, Rovito PF (2004). An approach to venous thromboembolism prophylaxis in laparoscopic Roux-en-Y gastric bypass surgery. Obes Surg.

[72] Minhem MA, Safadi BY, Habib RH (2018). Increased adverse outcomes after laparoscopic sleeve gastrectomy in older super-obese patients: analysis of American College of Surgeons National Surgical Quality Improvement Program Database. Surg Obes Relat Dis.

[73] Modasi A, Dang JT, Afraz S (2019). Bariatric surgery outcomes in patients on preoperative therapeutic anticoagulation: an analysis of the 2015 to 2017 MBSAQIP. Obes Surg.

[74] Nielsen AW, Helm MC, Kindel T (2018). Perioperative bleeding and blood transfusion are major risk factors for venous thromboembolism following bariatric surgery. Surg Endosc.

[75] Nimeri A, Mohamed A, El Hassan E (2013). Are results of bariatric surgery different in the Middle East? Early experience of an international bariatric surgery program and an ACS NSQIP outcomes comparison. J Am Coll Surg.

[76] Nudel J, Bishara AM, de Geus Susanna WL (2021). Development and validation of machine learning models to predict gastrointestinal leak and venous thromboembolism after weight loss surgery: an analysis of the MBSAQIP database. Surg Endosc.

[77] Obeid FN, Bowling WM, Fike JS (2007). Efficacy of prophylactic inferior vena cava filter placement in bariatric surgery. Surg Obes Relat Dis..

[78] Poulose BK, Griffin MR, Zhu Y (2005). National analysis of adverse patient safety events in bariatric surgery. Am Surg.

[79] Prasad P, Tantia O, Patle N (2012). An analysis of 1-3-year follow-up results of laparoscopic sleeve gastrectomy: an Indian perspective. Obes Surg.

[80] Prystowsky JB, Morasch MD, Eskandari MK (2005). Prospective analysis of the incidence of deep venous thrombosis in bariatric surgery patients. Surgery.

[81] Quebbemann BMD, Akhondzadeh MMD, Dallal RMD (2005). Continuous intravenous heparin infusion prevents peri-operative thromboembolic events in bariatric surgery patients. Obes Surg.

[82] Raftopoulos I, Martindale C, Cronin A (2008). The effect of extended post-discharge chemical thromboprophylaxis on venous thromboembolism rates after bariatric surgery: a prospective comparison trial. Surg Endosc.

[83] Ramly EP, Safadi BY, Aridi HD (2017). Concomitant removal of gastric band and gastric bypass: analysis of outcomes and complications from the ACS-NSQIP database. Obes Surg.

[84] Reames BN, Bacal D, Krell RW (2015). Influence of median surgeon operative duration on adverse outcomes in bariatric surgery. Surg Obes Relat Dis.

[85] Rezvani M, Sucandy I, Das R (2014). Venous thromboembolism after laparoscopic biliopancreatic diversion with duodenal switch: analysis of 362 patients. Surg Obes Relat Dis.

[86] Rezvani M, Sucandy I, Klar A (2014). Is laparoscopic single-stage biliopancreatic diversion with duodenal switch safe in super morbidly obese patients?. Surg Obes Relat Dis.

[87] Rodríguez JI, Kobus V, Téllez I (2020). Prophylaxis with rivaroxaban after laparoscopic sleeve gastrectomy could reduce the frequency of portomesenteric venous thrombosis. Ann R Coll Surg Engl.

[88] Scholten DJMDF, Hoedema RMMD, Scholten SE (2002). A comparison of two different prophylactic dose regimens of low molecular weight heparin in bariatric surgery. Obes Surg.

[89] Shah KG, Rajan D, Nicastro J (2012). Deep venous thrombosis in Lap Band surgery: a single center study. Indian J Surg.

[90] Sharma N, Chau WY (2020). Remodifying omentopexy technique used with laparoscopic sleeve gastrectomy: does it change any outcomes?. Obes Surg.

[91] Singh K, Podolsky ER, Um S (2012). Evaluating the safety and efficacy of BMI-based preoperative administration of low-molecular-weight heparin in morbidly obese patients undergoing Roux-en-Y gastric bypass surgery. Obes Surg.

[92] Spaniolas K, Kasten KR, Sippey ME (2016). Pulmonary embolism and gastrointestinal leak following bariatric surgery: when do major complications occur?. Surg Obes Relat Dis.

[93] Steele KE, Schweitzer MA, Prokopowicz G (2011). The long-term risk of venous thromboembolism following bariatric surgery. Obes Surg.

[94] Stroh C, Luderer D, Weiner R (2012). Actual situation of thromboembolic prophylaxis in obesity surgery: data of quality assurance in bariatric surgery in Germany. Thrombosis.

[95] Stroh C, Michel N, Luderer D (2016). Risk of thrombosis and thromboembolic prophylaxis in obesity surgery: data analysis from the German Bariatric Surgery Registry. Obes Surg.

[96] Surve A, Potts J, Cottam D (2022). The safety and efficacy of apixaban (Eliquis) in 5017 post-bariatric patients with 95.3% follow-up: a multicenter study. Obes Surg.

[97] Thereaux J, Lesuffleur T, Czernichow S (2018). To what extent does posthospital discharge chemoprophylaxis prevent venous thromboembolism after bariatric surgery?: results from a nationwide cohort of more than 110,000 patients. Ann Surg.

[98] Thereaux J, Veyrie N, Barsamian C (2014). Similar postoperative safety between primary and revisional gastric bypass for failed gastric banding. JAMA Surg.

[99] Westling A, Bergqvist D, Boström A (2002). Incidence of deep venous thrombosis in patients undergoing obesity surgery. World J Surg.

[100] Winegar DA, Sherif B, Pate V (2011). Venous thromboembolism after bariatric surgery performed by Bariatric Surgery Center of Excellence Participants: analysis of the Bariatric Outcomes Longitudinal Database. Surg Obes Relat Dis.

[101] Woo HD, Kim YJ (2013). Prevention of venous thromboembolism with enoxaparin in bariatirc surgery. J Korean Surg Soc.

[102] Young MT, Gebhart A, Phelan MJ (2015). Use and outcomes of laparoscopic sleeve gastrectomy vs laparoscopic gastric bypass: analysis of the American College of Surgeons NSQIP. J Am Coll Surg.

[103] Aminian A, Andalib A, Khorgami Z (2015). Who should get extended thromboprophylaxis after bariatric surgery? A risk assessment tool to guide indications for post-discharge pharmacoprophylaxis. Surg Obes Relat Dis.

[104] Nguyen NT, Hinojosa MW, Fayad C (2007). Laparoscopic surgery is associated with a lower incidence of venous thromboembolism compared with open surgery. Ann Surg.

[105] Gargiulo NJ, Veith FJ, Lipsitz EC (2007). The incidence of pulmonary embolism in open versus laparoscopic gastric bypass. Ann Vasc Surg.

[106] Khan S, Rock K, Baskara A (2016). Trends in bariatric surgery from 2008 to 2012. Am J Surg.

[107] Samuel I, Mason EE, Renquist KE (2006). Bariatric surgery trends: an 18-year report from the International Bariatric Surgery Registry. Am J Surg.

[108] Aiolfi A, Tornese S, Bonitta G (2019). Roux-en-Y gastric bypass: systematic review and Bayesian network meta-analysis comparing open, laparoscopic, and robotic approach. Surg Obes Relat Dis.

[109] Hassan M, Kerlakian G, Curry T (2008). Comparing outcomes of hand-assisted versus total laparoscopic gastric bypass. Surg Obes Relat Dis.

[110] Podnos YD, Jimenez JC, Wilson SE (2003). Complications after laparoscopic gastric bypass: a review of 3464 cases. Arch Surg.

[111] Buchwald H (2002). Overview of bariatric surgery. J Am Coll Surg.

[112] Kelleher DC, Merrill CT, Cottrell LT (2013). Recent national trends in the use of adolescent inpatient bariatric surgery: 2000 through 2009. JAMA Pediatr.

[113] Altieri MS, Yang J, Hajagos J (2018). Evaluation of VTE prophylaxis and the impact of alternate regimens on post-operative bleeding and thrombotic complications following bariatric procedures. Surg Endosc.

[114] Stein PD, Goldman J (2009). Obesity and thromboembolic disease. Clin Chest Med.

[115] Thorell A, MacCormick AD, Awad S (2016). Guidelines for perioperative care in bariatric surgery: Enhanced Recovery After Surgery (ERAS) Society recommendations. World J Surg.

[116] El Ansari W, El-Ansari K, Lock M (2023). Mind the overlap! Meta-analyses that synthesize the findings of primary studies based on large data registries: the case of metabolic and bariatric surgery. Obes Surg.

